# Appropriate normalization is critical to improve reproducibility of tissue ChIP-seq

**DOI:** 10.17161/sjm.v2i3.23692

**Published:** 2025-05

**Authors:** Qi Chu, Liu Peng, Lourdes Brea, Viriya Keo, Xiaodong Lu, Jindan Yu, Jonathan C. Zhao

**Affiliations:** 1 Department of Urology, Emory University School of Medicine, Atlanta, GA, USA;; 2 Department of Human Genetics, Emory University School of Medicine, Atlanta, GA, USA;; 3 Winship Cancer Institute, Emory University School of Medicine, Atlanta, GA, USA.; 4 Division of Hematology/Oncology, Department of Medicine, Northwestern University Feinberg School of Medicine, Chicago, IL, USA.

**Keywords:** Reproducibility, Chromatin-immunoprecipitation, tissue sample

## Abstract

Chromatin immunoprecipitation followed by next-generation sequencing (ChIP-seq) is a powerful technology for studying genetic and epigenetic regulation. However, ChIP-seq data can be heavily affected by variations in chromatin amount and composition, ChIP enrichment, library preparation, and sequencing depth, affecting its overall reproducibility across biological replicates. Here, we evaluated four ChIP-seq normalization methods utilizing triplicate Foxa1 ChIP-seq data performed in prostate cancer tissues from three mice. We found that count-per-million (CPM) normalization, although not affecting peak calling in individual samples, is very useful for visualization and comparison of peak distribution and intensity across samples. By contrast, equal-read normalization improves both peak identification and intensity comparison. Moreover, spike-in normalization took advantage of spike-in chromatin ChIP to correct technical variations in ChIP-seq, including ChIP enrichment, sample preparation, and sequencing. Lastly, input-adjusted spike-in normalization further accounts for differences in input chromatin amount across samples, which is especially crucial for tissue ChIP-seq that often starts with different amounts of input chromatin. Overall, our study demonstrated that appropriate normalization is essential to improve the reproducibility and comparability of ChIP-seq experiments and highlighted the importance of input-adjusted spike-in normalization for tissue ChIP-seq.

## Introduction

Chromatin immunoprecipitation coupled with next-generation sequencing (ChIP-seq) is a powerful technique for identifying genome-wide localization of transcription factors and histone modifications [1, 2]. It is common practice to compare ChIP-seq data across different experimental conditions, such as control vs. knockdown cells, to determine how transcription factor binding sites and epigenetic biomarkers are regulated [3]. However, such comparisons are often affected by variations in ChIP-seq experiments, including the quality and specificity of the antibody, the extent of ChIP enrichment, ChIP-seq library complexity, sequencing depth, and individual sample variations [4]. Data normalization is, therefore, essential to minimize technical biases and allow biologically meaningful discoveries.

The simplest and most commonly used normalization methods for ChIP-seq data are read-depth normalization, such as count-per-million (CPM) normalization and equal-read normalization. CPM normalization scales signals to a standard reference of one million reads, which is frequently utilized in heatmaps and average intensity plots to compare the intensity of ChIP-seq binding sites identified in various conditions. Equal-read normalization is used to bring all ChIP-seq samples in a comparing group to the same number of total reads, usually the lowest in the group, before peak discovery and intensity comparison. As both ChIP-seq peak number and intensity are positively correlated to the total number of reads before saturation, these normalization methods are useful to control for differences caused by different sequence depths.

In addition, different ChIP-seq experiments could have varying efficiency in ChIP enrichment, notoriously known for H3K27me3 ChIP-seq in control and inhibited cells [5]. To address such variations, spike-in normalization has been developed, which adds a known amount of exogenous spike-in chromatin, e.g. drosophila, to the sample chromatin, which was subjected to ChIP by a control spike-in antibody along with the experimental antibody, followed by concurrent sequencing [6]. During the subsequent data analyses, spike-in normalization will be used to scale reads of all samples by equalizing the spike-in reads. This method addresses common technical variations in ChIP-seq experiments, such as ChIP enrichment, PCR, library preparation, and sequencing.

The fundamental assumption of spike-in normalization is that the ratio between the amount of spike-in reads and sample reads remains constant across all samples based on the fact that an equal amount of spike-in chromatin was added to each sample chromatin during the experimentation. However, there are circumstances when the input DNA for a group of samples is different, due to variations in organ/tissue sizes, cell numbers/sizes, chromatin extraction efficiency, and other factors. It thus may be necessary to control for such input concentration differences by equalizing the ratio between spike-in DNA and sample DNA [7]. Input-adjusted spike-in normalization has been shown to improve the accuracy of detecting genome-wide differential modifications compared to equal-read normalizations and standard spike-in normalization [8]. Despite these advancements, the performance of different normalization strategies in tissue ChIP-seq has not been comprehensively evaluated.

In this study, we compared ChIP-seq normalization methods by analyzing triplicate Foxa1 ChIP-seq data from prostate cancer tissues derived from three mice. We evaluated how the different normalization methods affect peak calling and ChIP-seq intensity comparisons. Our findings highlight the superior performance of spike-in normalization and the importance of input adjustment in tissue ChIP-seq.

## Results

### Spike-in normalization out-performed CPM and equal-read normalization.

To evaluate the reproducibility of tissue ChIP-seq experiments, we dissected the whole prostates from three mice with prostate cancer, which have quite different prostate sizes and weights and thus varying total chromatin amounts (Table 1). Triplicate Foxa1 ChIP-seq experiments were performed using these three mouse prostate cancer tissues. To control for ChIP efficiency, we added an equal amount of spike-in DNA to each experiment. ChIP-seq revealed much fewer sample reads (4M) in replicate 1 (Rep 1), compared to the 24 and 23M unique sample reads for replicates 2 (Rep 2) and 3 (Rep 3), suggesting substantial differences among ChIP-seq experiments performed in tissues derived from different mice.

**Figure 1.**
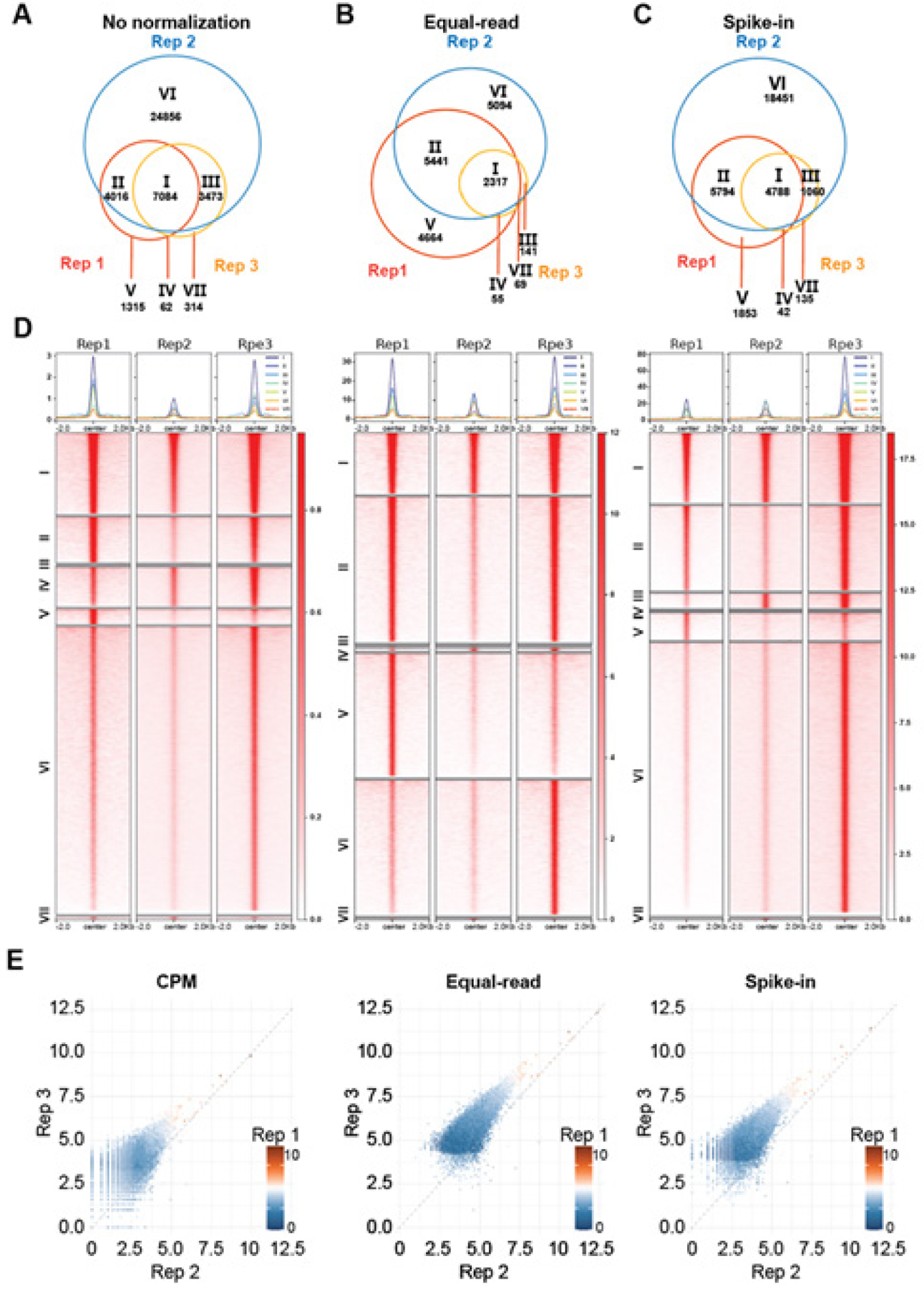
Traditional ChIP-seq normalization methods failed to correct systematic differences across biological replicates **A-C**. Venn diagrams showing the overlap of Foxa1 binding events among three biological replicates using the indicated normalization methods. **D**. Intensity plots and heatmap showing signal intensity of Foxa1binding events across three biological replicates. Signals are visualized within ±2 kb of the peak centers, with enrichment intensity represented by the color scale on the right. Peak clusters were defined as in A-C. **E**. Scatterplots of log-normalized peak signals across three biological replicates normalized by the indicated methods. The x- and y-axes represent the signal enrichment in Rep 2 and Rep 3, respectively, while Rep 1 is represented on the color scale.

To enable a fair comparison among the ChIP-seq experiments, we tested various normalization methods. We first identified Foxa1 binding peaks using the original sample read counts without any normalization (Fig. 1A). Surprisingly, we found Rep 1 and Rep 3 of 4M and 23M sample reads, respectively, yielded similar amounts (11–12k) of peaks, whereas substantially more peaks (>40k) were found in Rep 2, which had 24M sample reads. These data suggest that peak numbers were not only determined by the number of reads but by many other factors, even with the same antibody using the same protocol, indicating the complexity of tissue ChIP-seq. Further, Rep 1 and 3 peaks have the best (>60%) overlap, whereas Rep 2 has many peaks that were not reproduced in Rep 1 and 3, suggesting a high false-positive rate in Rep 2, likely due to poor ChIP-seq enrichment.

Next, to evaluate the potential effects of read counts on peak identification, we downsized all samples to the lowest read counts (4M of Rep 1), termed equal-read normalization (Fig. 1B). As expected, downsizing the read amount substantially reduced the number of peaks identified in Rep 2 and Rep 3, being consistent with the known correlation between read and peak numbers before saturation. There were much fewer overlapping peaks (2,317) after downsizing, suggesting false negatives, largely due to a huge loss of peaks in Rep 3. Almost all Rep 3 peaks were also detected in Rep 1 and Rep 2. This data suggests that Rep 3 has reasonable ChIP enrichment, which, however, has a weak overall signal and needs deep sequencing for peak discovery.

Due to such differential ChIP enrichment across the triplicates, we further attempted to use spike-in normalization to control for technical variations introduced during ChIP steps (Fig. 1C). As an equal amount of drosophila chromatin was added to each ChIP-seq experiment, we expect to see an equal proportion of drosophila reads relative to sample reads, which is the assumption used for spike-in normalization. Overall, spike-in normalization led to a down-sizing of Rep 2 and Rep 3, and thus fewer peaks were detected compared to those without normalization (Fig. 1A). On the other hand, it did not over-correct the data as equal-read normalization. There were thus more comparable numbers of peaks across replicates by spike-in normalization. Further, a significantly higher percentage of Rep 3 peaks were also detected by Rep 1 and 2 compared to no normalization, and significantly more Rep 1 peaks were reproduced by Rep 2 compared to equal-read normalization, supporting that spike-in normalization is superior to no normalization or equal-read normalization in reproducible peak identification.

Now that we have compared the impacts of the different normalizations on peak identification, we attempted to further investigate the peak intensity. We compared signal enrichment for all peaks identified in the three biological replicates using heatmaps, intensity plots, and scatterplots (Fig. 1D and 1E). For the peaks identified without normalization, CPM was utilized to normalize their read intensity during visualization, such as heatmaps and scatterplots. We observed an overall strong positive correlation among the samples – peaks with a strong signal in one sample also showed a strong signal in the other two samples, regardless of the normalization method (Fig. 1E). However, there was a systematic shift with Rep 3 peaks consistently having stronger enrichment than Rep 2 peaks, which was not corrected by any of the above normalization methods. The heatmaps and intensity plots further confirmed that, across all three normalization methods, peaks in Replicate 3 consistently showed stronger signal intensities than those in Replicate 2. However, the enrichment levels between replicates varied depending on the normalization method. Replicates 1 and 3 showed comparable enrichment following CPM and equal-read normalization, whereas Replicates 2 and 3 appeared more similar only when spike-in normalization was applied. This inconsistency suggests that different normalization strategies can lead to differing interpretations of signal similarity across samples (Fig. 1D).

### Input adjustment is required for ChIP-seq comparison across tissue samples

**Figure 2.**
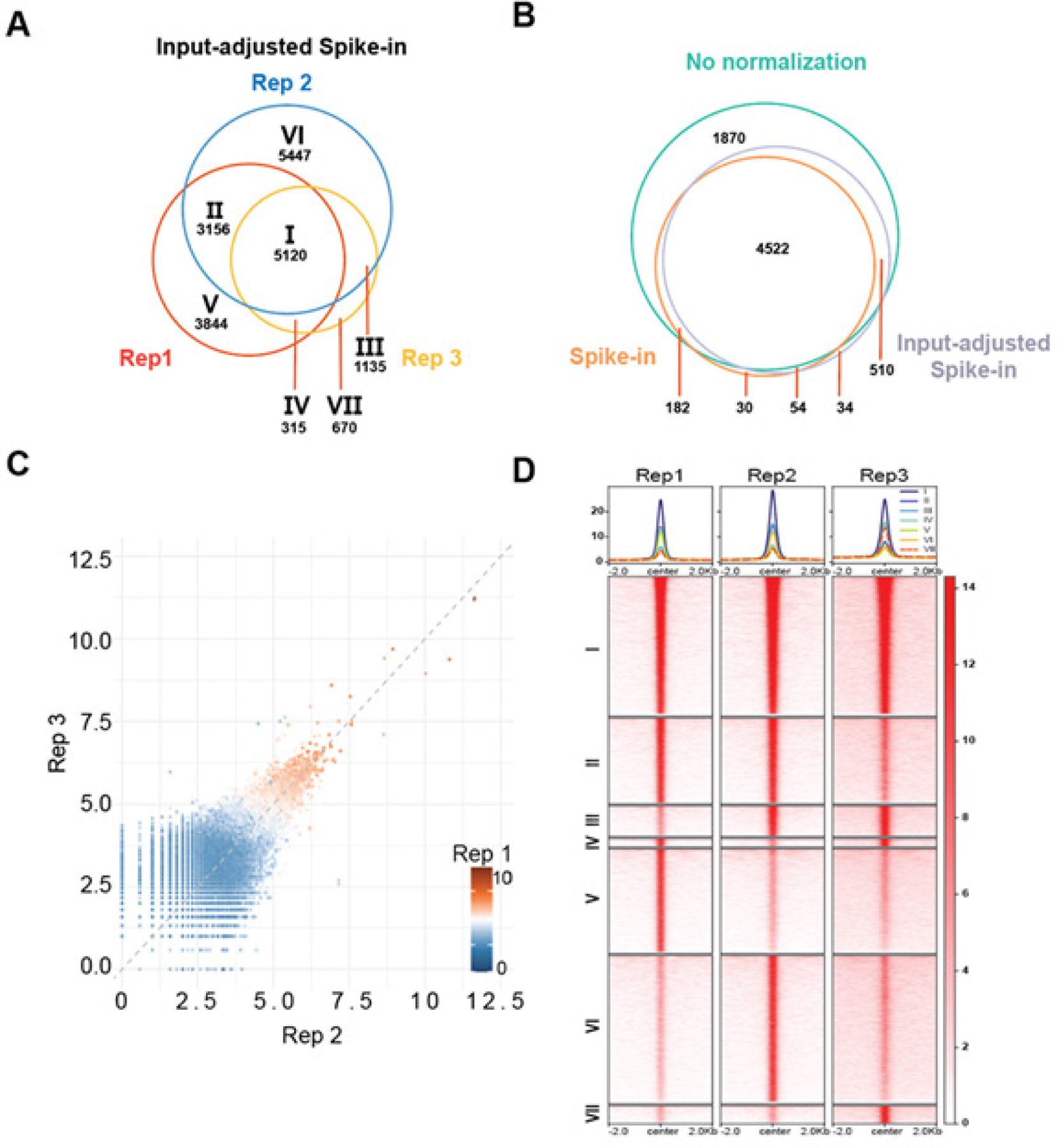
Input-adjusted spike-in normalization corrected systematic peak signal bias while improving reproducibility across biological replicates **A.** Venn diagrams showing the overlap of Foxa1 binding sites among three biological replicates using input-adjusted spike-in normalization. **B.** Venn diagrams showing the overlap of consensus Foxa1 binding sites that are shared by three biological replicates among three normalization methods. **C.** Scatterplots of log-normalized peak signals across three biological replicates. The x- and y-axes represent the signal enrichment in Rep 2 and Rep 3, respectively, while Rep 1 is represented on the color scale. **D.** Heatmap showing signal intensity for shared and unique Foxa1 binding events across three biological replicates. Signals are visualized within ±2 kb of the peak centers, with enrichment intensity represented by the color scale on the right. Peak clusters were defined as in A.

We suspect that the systematic differences in ChIP-seq signals between Rep 2 and 3 were related to the difference in prostate sizes and, thus, total chromatin amounts in the two mice. To account for this difference, we developed an input-adjusted spike-in normalization method. The assumption for this method is that the ratio of sample DNA to spike-in DNA should be equal to the ratio of sample reads to spike-in reads for each experiment. The normalized reads for each sample will be adjusted using a normalization factor (NF) as defined in the method section. First of all, peak identification algorithms detected a total of 5,120 overlapping Foxa1 binding events in triplicates, with much fewer Rep 2-only binding sites, indicating a better control of false positives caused by poor enrichment in Rep 2 (Fig. 2A). By comparing the consensus peaks in triplicates across CPM and traditional spike-in, the majority of consensus peaks called by input-adjusted spike-in normalization are also detected by either CPM or spike-in normalization with only 34 unique false-positive peaks, further supporting control of false positive (Fig. 2B). This performance is superior to CPM, equal-read, and spike-in normalization described earlier. Remarkably, scatter plot analyses revealed that input-adjusted spike-in normalization corrected the systematic differences in peak intensities such that the peaks were tightly distributed around the diagonal (Fig. 2C).

To further evaluate the ability and accuracy of input-adjusted spike-in normalization in distinguishing consistent Foxa1 peaks from ambiguous peaks caused by biological variance, (Fig. 2D). Analysis of peak cluster intensities revealed that the cluster of common peaks shared across all three replicates exhibited the highest intensity, indicating the most consistent and reliable Foxa1 peaks. In contrast, the replicate-specific peak clusters exhibited the lowest intensity, suggesting that these peaks represent biologically variable signals. Rep 2 has the most binding peaks, with a high number not captured by Rep 1 and 3, indicating potential false positives, which were limited to the smallest numbers by input-adjusted spike-in normalization. We identified these false-positive peaks as non-reproducible peaks that appeared only in Rep 2 and were called without any normalization. (Fig. S1A) (R2. Q5). By contrast, Rep 3 has specific but weak enrichment, and with this normalization method, it was able to capture a majority of the sites detected by Rep 1 and 2. These findings indicate that input-adjusted spike-in normalization effectively detected the reproducible Foxa1 binding sites and corrected systematic deviations in peak enrichment, thereby enhancing reproducibility and comparability across biological replicates.

## Discussion

Comparisons across ChIP-seq experiments done in different biological conditions are essential tools for studying genetic and epigenetic regulation. However, ChIP-seq experiments are inherently vulnerable to variations in chromatin amount and composition, ChIP enrichment, library preparation, sequencing depth, and technical variation. It is thus crucial to normalize ChIP-seq experiments to account for as many technical variabilities as possible before cross-sample comparisons for biological inferences. This is of utmost importance when comparing ChIP-seq experiments across tissue samples from different individuals.

In this study, we utilized triplicate Foxa1 ChIP-seq experiments in prostates derived from three mice with prostate cancer to evaluate four normalization approaches under conditions of significant technical variances. For example, Replicate 1 had markedly fewer uniquely mapped reads while the raw read count (23 M) and the amount of ChIP DNA (37 ng) appeared appropriate. A large number of unmapped reads (8 M) aligned to the salmon genome suggested contamination from magnetic beads blocked with sonicated salmon sperm DNA [9]. Additionally, poor antibody enrichment likely further contributed, as Replicate 1 yielded low amounts of immunoprecipitated chromatin and produced duplicate reads (11 M) reducing library complexity **(R2.Q2, R1.Q2)**. CPM normalization, while not affecting peak identification in each sample, is extremely useful for the visualization of multiple samples by scatter plots, heatmaps, and intensity plots to compare their ChIP-seq enrichment intensity. Equal-read normalization is not only useful for visualization but also calls peaks utilizing an equal number of reads for each sample in a comparing group, thus correcting for the positive impact of total read numbers on the number of ChIP-seq peaks and making peaks more comparable across samples [10]. Spike-in normalization took another step forward by rectifying sample ChIP enrichment efficiency using spike-in ChIP, further improving the reproducibility across replicate experiments.

However, none of the above normalization methods were able to account for systematic biases when input chromatin amount and complexity were different across samples. Standard spike-in normalization assumed that an equal amount of sample chromatin was utilized across experiments and thus added an equal amount of spike-in chromatin for each experiment. The subsequent data analyses were thus based on the assumption that the ratio between spike-in DNA and sample DNA remains constant across all samples, which, however, is often violated in tissue ChIP-seq experiments when chromatin amounts across individuals may vary significantly [11]. To this end, input-adjusted spike-in normalization introduced an additional correction by accounting for the variability in the ratio of spike-in DNA to sample input DNA, scaling the raw reads to equalize these ratios across samples[7]. We found that this approach enhanced peak-calling reproducibility and accurately corrected global signal variation.

In summary, our study compared the different ChIP-seq normalization methods and underscores the importance of applying appropriate normalization strategies in ChIP-seq data analysis. We found spike-in normalization superior to other methods when the input chromatin amount is the same across samples. In the event of varying input chromatin amounts, we found input-adjusted spike-in normalization very useful to account for differences in input concentrations across samples. Further, we recommend appropriate ChIP-seq data normalization before conducting downstream analyses, such as ChIP-seq peak calling and differential peak enrichment using consensus peaks identified across multiple biological replicates.

## Materials and Method

### ChIP-seq of mouse prostate tumors

ChIP-seq was performed following the previously described protocol (Lu et al., 2022). Briefly, 40mg of flash frozen 18wk timepoint prostate tumors were utilized for the Foxa1 ChIP experiments. Tissues were ground using an agate mortar and pestle and homogenized using the BeadBug benchtop homogenizer. Tissues were then double cross-linked with 2mM DSG for 10min, followed by 1% formaldehyde for 10min, at room temperature. Crosslinking was quenched with 0.125 M glycine for 5min at room temperature. Chromatin was fragmented using an E220 focused ultrasonicator (Covaris), 62.5 ng of Drosophila chromatin was added to each sample as spike-in DNA, and samples were pre-cleared with protein A agarose beads (Millipore) for 1hr. Then samples were incubated with the following antibodies overnight at 4C with rotation: 2 μg Foxa1 (Abcam, Cat#ab23738). 1 μg drosophila H3 antibody (Active motif, Cat# 61686) was also added per sample. Then, protein A agarose beads were added and incubated for 2h at 4C, followed by washing the beads with 1×dialysis buffer (2mM EDTA, 50mM Tris-Cl, pH 8.0) twice, and IP wash buffer (100mM Tris-Cl, pH 9.0, 500mM LiCl, 1% NP40, 1% deoxycholate) four times. Finally, protein-DNA complexes were eluted (50mM NaHCO3, 1% SDS), crosslinks were reversed, and DNA was purified using DNA Clean & Concentrator-5 kit (ZYMO Research). A total of 10 ng ChIP DNA was used to prepare libraries for ChIP-seq (NEB E7645L).

### ChIP-seq data analysis

For read quality filtering, duplicate reads were identified and removed by Picard (v3.0.0) and the adaptor reads removal process was performed with Trimmomatic V0.39. The reads were aligned against the mouse reference genome mm10 genome and D.melanogaster reference genome Dmel A4.10 respectively by Bowtie2 (v2.5.1). CPM normalization was performed with deepTools (v3.5.4) bam-Coverage by calculating the read counts within 20 bp windows as signal values, dividing by the total number of reads, and scaling the result to one million. Equal-read normalization was performed by downsampling the samples with the calculated normalization factor (NF) to the lowest reads sample with samtools (v1.17). The NF was calculated as $\text{Normalization}\;\text{Factor}=\frac{\text{min}(X)}{X_{i}}$, where *X*_*i*_ is the sample reads and min(*X*) is the sample with the lowest reads. The NF represents the proportion of reads that were randomly selected and retained from the input reads. The retained reads were counted within 20 bp windows as signal values by deepTools. Spike-in normalization was performed by counting D.melanogaster reads in each sample to calculate the down-sample normalization factor as $\text{Normalization}\;\text{Factor}=\frac{\text{min}(S)}{S_{i}}$ where *S*_*i*_ is D.melanogaster reads and min(S) is the sample with the lowest D.melanogaster reads. Down-sampling by NF was performed using samtools as previously described and reads within 20 bp windows were counted as signal values with deepTools. Input-adjusted spike-in normalization was performed by calculating the normalization factor as $\text{Normalization}\;\text{Factor}=\frac{C_{\text{mouse},i}/C_{\text{Drosophila},i}}{X_{i}/S_{i}},$ where *C*_*mouse,i*_ is input DNA concentration and *C*_*Drosophila,i*_ is Drosophila spike-in chromatin concentration, *X*_*i*_ is the sample read number and *S*_*i*_ is the drosophila read number for sample *i*. Samples were down sampled with samtools as previously described, and the signal values were calculated by counting reads in 20 bp windows with deepTools.

Tag directories were generated by HOMER (v4.11) from normalized reads for easier downstream peak calling and peak enrichment quantification. HOMER findPeaks was used for Foxa1 ChIP-seq narrow peak calling with default cutoff. Overlap of Foxa1 ChIP-seq peaks was determined with HOMER default setting, and Venn diagrams were generated with R script.

Bigwig files were generated with deepTools bam-Coverage with no additional normalization except for CPM normalization. Heatmaps were made with deepTools computeMatrix and plotHeatmap with the peaks clustered from previous peak overlap analysis. To evaluate the enrichment of the peaks, normalized signal around 1000bp peak center was quantified using HOMER annotatePeaks without additional normalization. The R package ggplot2 was used to generate Scatter plots.

## Supplementary Material

1

## Figures and Tables

**Table 1.** Triplicate Foxa1 ChIP-seq in 3 independent mouse prostate cancer tissues.

	M1661 (replicate 1)	M1672 (replicate 2)	M1666 (replicate 3)
Input DNA concentration (ng/μL)	29.6	17.3	35.6
Total input volume(μL)	20	20	20
Foxal antibody(μg)	2	2	2
Spike-in chromatin(ng)	62.5	62.5	62.5
Spike-in antibody(μg)	1	1	1
Total reads	23,323,662	35,733,071	37,907,791
Mapped reads	15,548,415	32,547,665	34,078,616
unique sample reads #	4,190,041	23,728,210	22,990,981
unique spike-in reads #	142,086	268,064	319,773
# sample reads with CPM normalization*	4,190,041	23,728,210	22,990,981
# sample reads after equal-read normalization	4,190,041	4,190,490	4,191,212
# sample reads after Spike-in normalization	4,190,041	12,576,184	10,217,507
# sample reads after Input-adjusted Spike-in normalization	4,190,041	4,639,325	11,376,353
# Peak with CPM normalization*	12,477	39,429	10,933
# Peak after equal-read normalization	12,477	12,993	2,582
# Peak after Spike-in normalization	12,477	30,093	6,025
# Peak after Input-adjusted Spike-in normalization	12,477	14,858	7,240

* CPM normalization only affects peak intensity in visualization

## References

[1] Johnson SS, Zhang C, Fromm J, Willis IM, Johnson DL: Mammalian Maf1 is a negative regulator of transcription by all three nuclear RNA polymerases. Mol Cell 2007, 26(3):367–379; doi:10.1016/j.molcel.2007.03.021. 17499043

[2] Mikkelsen TS, Ku M, Jaffe DB, Issac B, Lieberman E, Giannoukos G, Alvarez P, Brockman W, Kim TK, Koche RP : Genome-wide maps of chromatin state in pluripotent and lineage-committed cells. Nature 2007, 448(7153):553–560; doi. 17603471 10.1038/nature06008 PMC2921165

[3] Bardet AF, He Q, Zeitlinger J, Stark A: A computational pipeline for comparative ChIP-seq analyses. Nat Protoc 2011, 7(1):45–61; doi:10.1038/nprot.2011.420. 22179591

[4] Landt SG, Marinov GK, Kundaje A, Kheradpour P, Pauli F, Batzoglou S, Bernstein BE, Bickel P, Brown JB, Cayting P : ChIP-seq guidelines and practices of the ENCODE and modENCODE consortia. Genome Res 2012, 22(9):1813–1831; doi:10.1101/gr.136184.111. 22955991 PMC3431496

[5] Fong KW, Zhao JC, Lu X, Kim J, Piunti A, Shilatifard A, Yu J: PALI1 promotes tumor growth through competitive recruitment of PRC2 to G9A-target chromatin for dual epigenetic silencing. Mol Cell 2022, 82(24):4611–4626 e4617; doi:10.1016/j.molcel.2022.11.010. 36476474 PMC9812274

[6] Bonhoure N, Bounova G, Bernasconi D, Praz V, Lammers F, Canella D, Willis IM, Herr W, Hernandez N, Delorenzi M : Quantifying ChIP-seq data: a spiking method providing an internal reference for sample-to-sample normalization. Genome Res 2014, 24(7):1157–1168; doi:10.1101/gr.168260.113. 24709819 PMC4079971

[7] Vale-Silva LA, Markowitz TE, Hochwagen A: SNP-ChIP: a versatile and tag-free method to quantify changes in protein binding across the genome. BMC Genomics 2019, 20(1):54; doi:10.1186/s12864-018-5368-4. 30654749 PMC6337847

[8] Patel LA, Cao Y, Mendenhall EM, Benner C, Goren A: The Wild West of spike-in normalization. Nat Biotechnol 2024, 42(9):1343–1349; doi:10.1038/s41587-024-02377-y. 39271835 PMC12266361

[9] McCullough SD, On DM, Bowers EC: Using Chromatin Immunoprecipitation in Toxicology: A Step-by-Step Guide to Increasing Efficiency, Reducing Variability, and Expanding Applications. Curr Protoc Toxicol 2017, 72:3 14 11–13 14 28; doi:10.1002/cptx.22. PMC6142182 28463415

[10] Orlando DA, Chen MW, Brown VE, Solanki S, Choi YJ, Olson ER, Fritz CC, Bradner JE, Guenther MG: Quantitative ChIP-Seq normalization reveals global modulation of the epigenome. Cell Rep 2014, 9(3):1163–1170; doi:10.1016/j.celrep.2014.10.018. 25437568

[11] Wu D, Wang L, Huang H: Protocol to apply spike-in ChIP-seq to capture massive histone acetylation in human cells. STAR Protoc 2021, 2(3):100681; doi:10.1016/j.xpro.2021.100681. 34337446 PMC8313745

