# Supplementary material for: Appropriate normalization is critical to improve reproducibility of tissue ChIP-seq
Source: Serican J Med. Author manuscript; Available in PMC 2026 Feb 7. (PMC12880172; doi:10.17161/sjm.v2i3.23692)

Figure S1. Poor ChIP-seq enrichment induced false-positive Foxa1 binding sites, Related to Figure 1.
A Genome browser tracks showing false-positive Foxa1 binding site (highlighted in yellow) and true-positive Foxa1 binding site (highlighted in red)

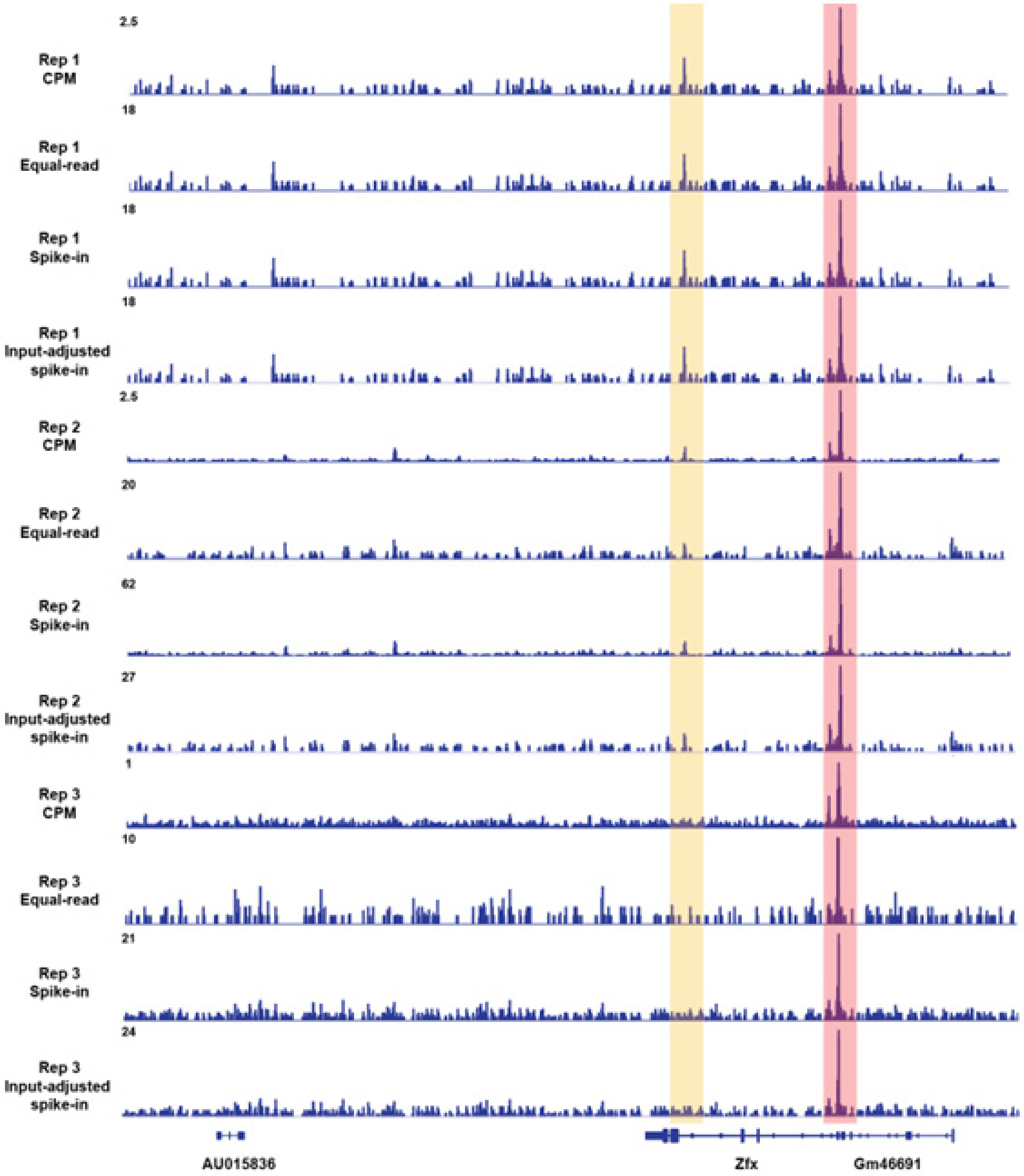

Supplement: 1 [file NIHMS2141244-supplement-1.pdf]
